# Emerging Personalized Opportunities for Enhancing Translational Readthrough in Rare Genetic Diseases and Beyond

**DOI:** 10.3390/ijms24076101

**Published:** 2023-03-23

**Authors:** Roland N. Wagner, Michael Wießner, Andreas Friedrich, Johanna Zandanell, Hannelore Breitenbach-Koller, Johann W. Bauer

**Affiliations:** 1 Department of Dermatology and Allergology, University Hospital of the Paracelsus Medical University, 5020 Salzburg, Austria; 2 Department of Biosciences, University of Salzburg, 5020 Salzburg, Austria

**Keywords:** translational readthrough, premature termination codon (PTC), nonsense suppression therapy, epidermolysis bullosa (EB)

## Abstract

Nonsense mutations trigger premature translation termination and often give rise to prevalent and rare genetic diseases. Consequently, the pharmacological suppression of an unscheduled stop codon represents an attractive treatment option and is of high clinical relevance. At the molecular level, the ability of the ribosome to continue translation past a stop codon is designated stop codon readthrough (SCR). SCR of disease-causing premature termination codons (PTCs) is minimal but small molecule interventions, such as treatment with aminoglycoside antibiotics, can enhance its frequency. In this review, we summarize the current understanding of translation termination (both at PTCs and at cognate stop codons) and highlight recently discovered pathways that influence its fidelity. We describe the mechanisms involved in the recognition and readthrough of PTCs and report on SCR-inducing compounds currently explored in preclinical research and clinical trials. We conclude by reviewing the ongoing attempts of personalized nonsense suppression therapy in different disease contexts, including the genetic skin condition epidermolysis bullosa.

## 1. Introduction

Heritable changes to nuclear DNA, such as deletions, insertions or point mutations, account for a majority of genetic disorders [1]. Point mutations can lead to silent mutations, with no apparent effect, or they can result in pathogenic missense or nonsense mutations. The latter generate an unscheduled stop signal, termed a premature termination codon (PTC), in the open reading frame of an affected gene and mRNA, respectively. Upon mRNA translation, this delivers a truncated, nonfunctional protein.

In recent decades, multiple approaches have been pursued to alleviate the severity of genetic diseases, most often rare diseases, caused by nonsense mutations. Growing evidence suggests that pharmacological agents that promote the translational bypassing of PTCs (also called nonsense suppression) constitute a promising solution, allowing the production of full length, functional proteins at levels sufficient to improve disease phenotypes [2,3,4,5]. Importantly, the respective drugs may often be applicable for systemic use. The clinical applicability of nonsense suppression depends on multiple factors, such as substance class used, the identity of the stop codon, 5′ and 3′ sequences immediately surrounding the stop codon and multiple other molecular factors [6,7,8,9,10,11,12,13,14]. So far, the vast majority of interventional clinical trials on nonsense suppression investigated substances classified as aminoglycoside antibiotics or the non-aminoglycoside ataluren (see Supplementary Table S1). However, problematic side effects caused by prolonged usage hampered the further development of aminoglycosides in clinical settings [15]. In addition, the evaluation of results obtained in clinical studies often proved challenging, since patient groups in rare genetic diseases are frequently composed of highly diverse genetic backgrounds, making general conclusions on treatment efficiencies difficult. Consequently, nonsense suppression should be considered to be a targeted personalized medication that takes into account both the genetic phenotype of each individual patient and the architecture of the mRNA species that harbors the PTC. In this review, we describe the mechanisms involved in the recognition of PTCs and the bypassing of PTCs by translational readthrough and report on compounds currently explored in preclinical research and clinical trials for the treatment of various diseases caused by PTCs. We highlight recently discovered pathways that influence PTC-readthrough and how these could be exploited for the development of improved nonsense suppression therapies. We conclude by summarizing the ongoing attempts of nonsense suppression therapy in the context of various diseases, including the genetic skin disease epidermolysis bullosa.

## 2. Molecular Mechanisms of Translation Termination and Nonsense Suppression

### 2.1. Natural and Induced Stop Codon Readthrough

The termination of mRNA translation is usually triggered when a stop codon, both cognate stop codons and PTCs, enters the ribosomal A site with release of the growing polypeptide chain from the peptidyl-tRNA in the P site [16]. However, both the fidelity of decoding sense codons and stop codons is not 100% efficient [9] and amino-acid misincorporations during translation have been reported to occur once in every 1000 to 10,000 codons [17]. In the case of a stop codon, this fidelity drawback, i.e., the ability of a ribosome to continue translation past a stop codon is called stop codon readthrough (SCR) [18,19]. Indeed, SCR is a natural process exploited to expand translational space and to allow for greater protein diversity [20,21]. This results in the production of C-terminally extended proteins that, compared to their canonical counterparts, often serve different physiological functions or display altered subcellular localizations. SCR (natural and induced) depends on multiple factors, such as the nature of the stop codon (UAA, UAG or UGA), sequence context (nucleotides preceding and succeeding the stop codon), mRNA structure, elongation factors and the absence or presence of stimulating compounds [6,7,8,9,11,12]. For example, when a cytosine is found at the +4 position (the nucleotide immediately following the stop codon), basal readthrough levels are increased [6,7,11]. Additionally, other positions surrounding the stop codon seem to have an influence on readthrough levels [8], as does the secondary structure of the mRNA [18,22,23]. Interestingly, mRNA modification mediated by the replacement of uridine with pseudouridine (Ψ) at PTCs (e.g., UAA→ΨAA) can promote SCR [24]. However, the level of SCR in mammalian cells seems context-dependent and negligible under most circumstances [25]. Natural SCR is driven by simple or complex sequence motives, termed readthrough elements, located at 5′ and 3′ of the stop codon, with the four downstream base pairs being particularly important [21]. Some of these elements include complex, higher-order RNA secondary structures (e.g., stem-loops) [23]. Although most prevalent in viral genomes, there is evidence for physiological readthrough of normal stop codons in a growing number of organisms, including bacteria, yeast, insects, humans and, most recently, in *Arabidopsis thaliana* [20,21,26,27,28,29]. Using ribosome-profiling data, evidence for stop codon readthrough was found in 144 *A. thaliana* genes [29]. Constituting an important expansion of the proteome, the C-terminally extended isoforms often contained putative nuclear localization signals or transmembrane helices that influence the function, localization and stability of the respective proteins [28,30]. For a number of *Drosophila* genes, the relative abundance of the two isoforms is not only tissue-specific but changes through different developmental stages. Tissue-specific differences in the relative abundance of factors that modulate translation termination (e.g., termination factor eRF1 splice variants) control such differential translational readthrough [31]. Interestingly, the readthrough of the canonical stop codon in human *AQP4X* generates a C-terminally elongated isoform that is exclusively located perivascularly and was recently shown to be implicated in the clearance of amyloid-β from the brain [32,33].

Considering that PTCs caused by pathogenic nonsense mutations constitute an important subgroup of stop codons, SCR (specifically of PTCs) would often be highly desirable. Induced SCR (or nonsense suppression) seeks to increase the incorporation rates of near-cognate tRNAs at a stop codon triplet [34]. SCR can lead to the insertion of an alternative set of amino acids. In addition, when SCR is triggered by lowering translational accuracy, it lowers the fidelity of translation and, consequently, also impacts the decoding of sense codons [10]. A well-known class of drugs that can magnify SCR are aminoglycoside antibiotics [35,36,37,38,39]. These drugs manipulate decodin by binding to the ribosomal decoding site, thereby changing translational accuracy. Although aminoglycosides were first developed to target the prokaryotic ribosome to compromise the faithful protein synthesis of bacterial pathogens, they also bind, with lesser affinity, to the eukaryotic ribosome [39]. Binding to the eukaryotic ribosome constitutes their therapeutic potential as translational-readthrough-inducing drugs (TRIDs) [40]. Aminoglycosides (and ideally any TRID) would favor the readthrough of PTCs, before nonsense mediated decay (NMD) is able to degrade the PTC mRNA, thereby reducing the substrate for TRID intervention. In mammalian mRNAS, such a signal would be the exon junction complex (EJC), which travels with the newborn mRNA to a first round of translation, where the integrity of an mRNA is monitored. Inevitable, treatment with most, if not all, TRIDs tested so far also increases the readthrough of the natural stop codons, indicated by an accumulation of ribosome-protected fragments (RPFs) in the 3′ region of many mRNAs [11]. This results in the production of proteins that have C-terminal extensions [41]. Moreover, since translational accuracy is primarily influenced during decoding, TRIDs can also promote the incorporation of non-cognate (or near-cognate) amino acids, interfering with protein integrity. This misreading could also lead to so-called translation error clusters, which are responsible for putative side effects [42]. Another common side effect of aminoglycosides, especially at high dosage and prolonged use, is the loss of hearing [43], as this class of drugs targets fast dividing cells of the inner ear (and their mitochondria) the most. Even though aminoglycosides (and TRIDs in general) are potent inducers of PTC readthrough, the exact mechanism of action frequently seems quite difficult to understand. It is often unknown whether these drugs only lower translational accuracy by distorting the ribosomal decoding site, influence elongation rates or target other cellular pathways that influence the translatome of the cell as a whole. Efforts to improve the utility of aminoglycosides as TRIDS are ongoing [43,44,45,46].

To what extend readthrough therapies are suited to correct the consequences of splice-site mutations in addition to PTCs remains to be fully clarified. However, studies on proximal spinal muscular atrophy (SMA) indicate that nonsense suppression (induced by treatment with aminoglycosides) of alternatively spliced survival motor neuron 2 (*SMN2*) transcripts leads to the extension and stabilization of the SMN protein, elevated SMN protein levels and improved motor function in SMA mice in vivo [47,48,49,50]. SMA is caused by *SMN1* loss-of-function mutations. *SMN2* is identical to *SMN1*, except for a C-to-T transition in exon 7 that leads to the loss of a splicing enhancer. The resulting *SMN2* transcript lacks exon 7, harbors a PTC in exon 8 and leads to the production of an unstable SMN protein.

### 2.2. Translation Termination and Nonsense-Mediated Decay

An important strategical aspect of induced SCR will consider the capacity of the translation machinery to differentiate between normal termination codons (NTCs) and PTCs [51]. In particular, for mammalian mRNAs, the ribosome differentiates the encounter of a PTC versus that of an NTC mainly by the absence or presence of the EJC [52,53]. While for NTCs, no EJCs can be found downstream of the stop codons, for PTCs downstream, EJCs are present. This leads to an interaction of the ribosome stalled at the PTC with the EJC, and this in turn triggers the termination of translation by nonsense-mediated RNA decay (NMD) [54,55,56]. The disruptive translation of a PTC-bearing transcript, however, promotes the production of a truncated protein that can no longer fulfils its intrinsic function. Alternatively, a shortened, dominant-negative version of the respective protein that is damaging to the cell or the organism may be produced. As defective messages (containing frameshifts and PTCs) not only result from mutational events but also frequently arise from aberrant splicing events, NMD has evolved as an evolutionarily conserved surveillance mechanism to identify defective transcripts and mark them for rapid degradation before their translation can exert potentially detrimental effects. As a drawback, the permanent removal of these transcripts from the mRNA pool makes them no longer available for induced SCR. Therefore, translation into functional full-length proteins is minimized and renders the cell permanently deficient for the affected protein. So, while the NMD pathway mitigates the potentially harmful properties of dysfunctional proteins, resulting from the translation of PTC-bearing transcripts, it can substantially impede any prospects for efficient nonsense suppression therapies [57].

**Figure 1:**
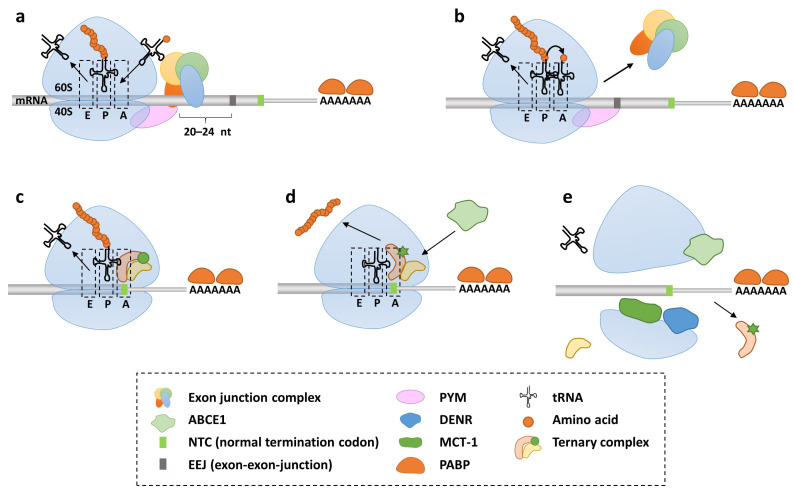
Translation termination at normal termination codons. (**a**) During translation, the ribosome (assembled from a 60S and a 40S subunit) transverses the capped and polyadenylated mRNA to translate it into a polypeptide. Incoming aminoacyl-tRNAs decode the mRNA and are accommodated in the A-site. The P-site hosts the peptidyl tRNA and during elongation by peptidyl transferase activity transfers the growing peptide chain onto the incoming amino acid of the A-site tRNA. During translocation, the elongated A-site tRNA moves into the P-site, while the bare P-site tRNA leaves the ribosome via the E-site. Exon junction complexes (EJC comprises eIF4AIII, Y14, MLN51 and MAGOH) decorate the mRNA approximately 20–24 nucleotides upstream of each exon–exon-junction (EEJ). PYM associates with the small ribosomal subunit and mediates interaction with EJC components. (**b**) As the ribosome moves along the mRNA during the pioneer round of translation, EJCs are removed along the mRNA and the ribosome arrives at the normal termination codon (NTC). (**c**) Upon encountering an NTC, the ternary termination complex (composed of eukaryotic release factors eRF1 and eRF3 activated by GTP) enters the A-site. (**d**) After stop codon recognition and GTP hydrolysis, the polypeptide is released from the peptidyl-tRNA located in the P-site. (**e**) Ribosome recycling post-termination is initiated by the ATP-binding cassette sub-family E member 1 (ABCE1) to dissociate the subunits. Multiple copies in T-cell lymphoma-1 (MCT-1) together with density-regulated protein (DENR) promote the dissociation of deacylated tRNA and mRNA from the 40S subunit.

In eukaryotes, every mRNA transcribed is subject to a number of pre-processing steps for competent export from the nucleus to the cytosol. Those include (1) the capping of the 5′-end with a methylguanosin-moiety as a platform for assembly of the cap binding complex (CBC), consisting of proteins CBP80 and CPB20; (2) the polyadenylation of the 3′-end; and (3) the coordinated removal of introns from pre-mRNA mediated by the spliceosome [58,59,60]. During splicing, the spliceosome deposits a multi-subunit protein complex, the EJC, with its core members eIF4AIII, Y14, MLN51, and MAGOH, approximately 20–24 nucleotides upstream of each processed splice junction [61,62,63] (Figure 1a). Following nuclear export and dependent on the presence of the CBC, each new messenger ribonucleoprotein (mRNP) is delegated to a pioneer round of translation [52,64,65]. Ribosome recruitment required for pioneer initiation of protein synthesis is guided by the specific interaction between CBP80/20-dependent translation initiation factor (CTIF) and eIF3G, a component of the eukaryotic translation initiation factor 3 (eIF3) complex [66]. During the pioneer round of translation, the small 40S subunit of the translating ribosome associates with the C-terminal domain of disassembly factor PYM that senses the presence of EJC bound components MAGOH-Y14 via its N-terminal domain and aids in the displacement of all EJCs the ribosome encounters [53,67] (Figure 1b). Translation termination is then triggered when the ribosome reaches one of the normal termination codons UAG, UGA or UAA in the A-site, unless it runs into a PTC first [68] (Figure 1c). Translation termination in eukaryotes requires the two eukaryotic release factors eRF1 and eRF3 [69]. The class I release factor eukaryotic release factor 1 (eRF1) is a tRNA-mimic which recognizes all three stop codons, whereas in prokaryotes, the class I release factor RF1 recognizes UAA and UAG and RF2 recognizes UAA and UGA [70]. In both organismal domains, the class II release factor eRF3 is a GTP binding protein that facilitates the termination process [68,71]. The ternary complex composed of eRF1/3 loaded with GTP enters the A-site of the ribosome. After stop codon recognition by domain 1 of the eRF1 subunit and GTP hydrolysis by eRF3, eRF1 mediates the hydrolysis of the polypeptide from the peptidyl-tRNA located in the P-site, the release of the protein product and the later recycling of the post-termination complex [72,73,74,75] (Figure 1d). The binding between the ribosome and the eRF1/eRF3-GTP ternary complex is further enhanced by the presence of poly(A)binding protein (PAPB) tethered in the 3′-poly(A) region [76]. Previous studies show that an important function of PABP is to protect poly(A)-mRNAs from decay by cellular nucleases [77]. In addition to stop codon recognition by eRF1, the efficiency of termination can be positively influenced by the presence of a purine residue at the +4 position, as well as by a purine at the +5 position if a pyrimidine already occupies the +4 position [68,78]. The recycling of the post-termination complex includes the dissociation of the 80S ribosome mediated by ATP-binding cassette sub-family E member 1 (ABCE1) into the 60S large and 40S small core-subunits, where the peptidyl-tRNA and the translated mRNA still remain associated with the small subunit [79] (Figure 1e). Then, the release of the deacylated tRNA and the mRNA are initiated through the eukaryotic translation initiation factors eIF3, eIF1, eIF1A and eIF3J [80]. In addition, eIF2D (ligatin) and multiple copies in T-cell lymphoma-1 (MCT-1) together with density-regulated protein (DENR), further promoting the dissociation of deacylated tRNA and mRNA from 40S subunits, notwithstanding their function in translation initiation [81]. Finally, after the completion of the pioneer round of translation, the CBC is replaced by eukaryotic translation initiation factor 4E (eIF4E) and the mRNA is ready to be loaded onto the ribosome for steady-state translation and is no longer available for further CBC-initiated pioneering rounds of translation [54,82]. Importantly, efficient ribosome recycling enables ribosomes and mRNAs to participate in multiple rounds of translation.

**Figure 2:**
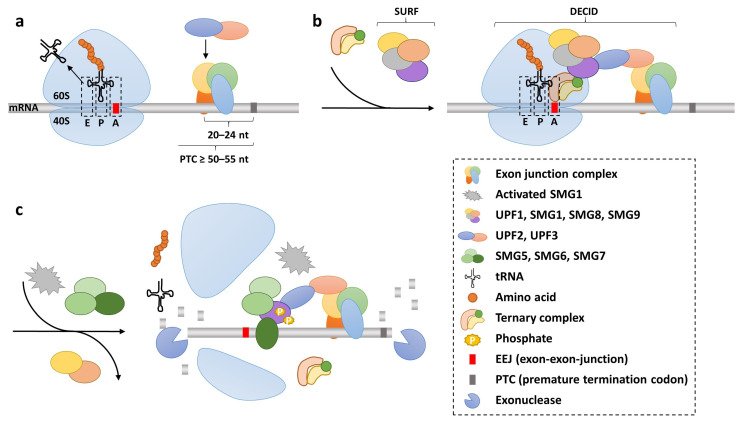
Premature termination of translation and initiation of nonsense mediated decay. (**a**) The ribosome is halted by the premature termination codon (PTC) motive located at least 50–55 nt upstream of an exon–exon junction (EEJ) and this is reinforced by an exon junction complex. The ternary complex enters the A-site of the stalled ribosome, and UPF2 and UPF3 are recruited to the exon junction complex (EJC). The readthrough propensities of different PTCs (UGA, UAG or UAA) as well as the nucleotide immediately succeeding the stop codon (+4 nt) are indicated (green indicates lowest stringency; red indicates highest stringency of translation termination). (**b**) The SURF (SMG1–UPF1–eRF1–eRF3) complex closes the gap between the paused ribosome and the EJC, forming the so-called DECID complex. (**c**) Release of SMG8 and SMG9 from the SURF complex leads to the activation of SMG1, phosphorylation of UPF1, the release of eRF1/eRF3 and the dissociation of ribosomal subunits. Recruitment of decay factors SMG5, SMG6 and SMG7 mediates mRNA nucleolytic digestion.

When a nonsense mutation is located in the open reading frame of a protein-coding gene, it leads to the formation of a PTC and, consequently, to the activation of NMD [83]. It is noteworthy that the mere presence of a PTC within a protein-coding transcript is not sufficient to stimulate its degradation through NMD. Instead, the main trigger of NMD seems to be the aberrant and inefficient process of translation termination at PTCs compared to translation termination at NTCs [83]. Central to NMD are factors of the up-frameshift protein (UPF) family, as it was reported that deletion or suppression of UPF genes eliminates NMD and prevents the decay of PTC-bearing transcripts in eukaryotes [84,85]. Here, it is important to note that decay factors UPF2 and UPF3 are only detected on mRNA bound by CBC but not mRNA bound by eIF4E. These and other data indicate that NMD targets CBC-bound mRNA during a pioneer round of translation while leaving eIF4E-bound mRNA insusceptible against NMD [54,64,82]. The major decay factor UPF1 itself does not seem to act exclusively on nonsense transcripts; furthermore, UPF1 shows a translation independent affinity for mRNAs and is stripped of it by the ribosome [86]. This would allow UPF1 to be rapidly activated to trigger further NMD processes when the ribosome stalls at a PTC [86]. However, NMD controls the quality of mRNAs and ensures that mutant transcripts (resulting from mutations or erroneous splicing) with shortened reading frames are detected early and targeted for degradation before steady state translation leads to C-terminally truncated proteins that could have deleterious functions and may permanently disrupt cellular homeostasis [57]. However, NMD is also not 100% efficient and varies among cells, indicating that the level of NMD efficiency is an inherent characteristic of different cell types [87]. This results in rare cases where PTC mRNAs (the targets of TRID intervention) evade NMD-mediated degradation and small amounts of defective protein are translated from faulty transcripts [88]. How the NMD mechanism discriminates between NTCs and PTCs is still content of an ongoing debate. The EJC-dependent model assumes that, during the pioneering round of translation, when the ribosome encounters a PTC located at least 50–55 nucleotides upstream of an exon–exon junction site, which is occupied by an EJC, the conditions to trigger NMD are met, since this situation would suggest an EJC located downstream of a termination codon [52,82] (Figure 2a). The paused ribosome with the PTC located in the A-site accommodates the binding of eRF1, which is part of the SURF complex that further consists of eRF3, the PI3K (phosphatidylinositol 3-kinase)-related protein kinase SMG-1 (suppressor with morphogenetic effects on genitalia), the kinase activity of which on UPF1 is initially suppressed by SMG8/SMG9 [89,90,91] (Figure 2b). Interaction between the SURF-complex and the downstream EJC, a formation termed the decay-inducing complex (DECID), leads to the release of SMG8/SMG9 through the interplay between UPF2 and SMG8, which finally activates SMG1 and permits the phosphorylation of UPF1, the release of eRF1/eRF3 and the ribosome [89,90] (Figure 2c). Phosphorylated UPF1 is the major trigger for NMD with the subsequent recruitment of the RNA decay factors SMG5/SMG7, which enable SMG6 endonuclease activity and ultimately initiate RNA degradation [89,90,92,93].

### 2.3. Reporter Assays and Detection of Readthrough at Genomic Scale

In order to find new readthrough drugs, reporter based assays are commonly used in research. Those assays have certain limitations when related to clinical application. One of the questions, which have to be addressed, is which amino acid is inserted at the position of the PTC, and whether the resultant protein is functional [94]. Since most reporter-based assays are established in a certain cell line, there is always the question of translatability between the readout of the assay and the actual level of protein being produced in patient-derived cells or patients during clinical trials. Since the level of readthrough depends on multiple factors (as discussed above), every assay should ideally by adjusted to specific disease backgrounds. Even if preclinical experiments unequivocally indicate that the readthrough of a certain PTC seems therapeutically promising, the effective concentrations of aminoglycoside antibiotics (or other compounds) that are necessary for sufficient SCR cannot be realistically realized in patients’ sera during long-term treatment regimens. Fine-tuning the therapeutic window for each drug and mitigating the side effects of prolonged treatments are major challenges in the development of next-generation TRIDs.

An important starting point for the discovery and evaluation of new translational readthrough-inducing compounds is the implementation of highly sensitive SCR detection methods, ideally at genome-scale. Standard methods for the detection of SCR, such as Western blotting or reporter-based assay, do not provide the necessary resolution and cannot be scaled to genomic levels. Luciferase-based reporter assays provide good sensitivity for detecting readthrough events and are widely adapted for evaluating existing compounds and high-throughput screening efforts to identify novel compounds. Reporter assays only allow the readthrough assessment of singular PTCs (one PTC at a time). Induced SCR, however, is not limited to the particular PTC-bearing gene of interest but affects normal stop codons as well as physiological NMD targets (produced by spontaneous mutations or aberrant splicing) on a global, genomic scale. The relatively new next generation sequencing (NGS) method ribosome profiling (Ribo-seq) is capable of estimating readthrough on a global scale [95]. The basic principle behind this method is that the translating ribosome can be stopped through the application of an elongation inhibitor, i.e., cycloheximide. The position where the stopped ribosome resides protects an RNA stretch of approximate 30 nucleotides in length from RNAse digestion. These ribosome protected footprints (RPF) are purified through column filtration, the subtraction of rRNA contaminations and gel electrophoresis, following a well-established protocol. RPFs are then subjected to NGS library preparation and sequenced on a high-throughput sequencer [96]. After bioinformatic pre-processing and quality control of the reads, mapping against a genome assembly with a splice-aware alignment software or against a transcriptome delivers the position of each read, i.e., the ribosomal position it derived from [97]. The valuable data derived from the positions and the respective counts allow for the analysis of a broad range of parameters, such as triplet-periodicity (the movement of the ribosome in three nucleotide steps along a transcript), the discovery of translation in previously noncoding regions and translational efficiency if matched RNA-seq data are available (routinely applied together with Ribo-seq). However, the bottleneck for the broader application of Ribo-seq is the lack of a standardized wet-lab protocol and bioinformatic analysis pipeline that makes it harder to reproduce and compare results. Therefore, a first effort by a large consortium established a framework for the standardized annotation of translated open reading frames, which was recently published [98]. In a SCR-related context, Ribo-seq offers the possibility to detect reads that derive from readthrough events at PTCs as well as NTCs. Reads mapped downstream of PTCs indicate PTC-readthrough. Reads mapped downstream of NTCs reflect the presence of ribosome in the 3′-untranslated region of the respective genes. An ideal readthrough-inducing compound would on the one hand promote high levels of full-length protein and, on the other hand, display high specificity to distinguish between PTCs and NTCs. Here, Ribo-seq offers the possibility of comprehensive evaluation, since it enables the detection of readthrough events in larger detail, as demonstrated previously. Wangen et al. introduced this method to demonstrate global SCR upon the application of aminoglycoside antibiotics. Furthermore, the use of the potent TRID, the aminoglycoside G418 (geneticin), alters gene expression to a large extent [11].

## 3. Induction of Nonsense Suppression

### 3.1. Aminoglycoside Antibiotics

**Figure 3:**
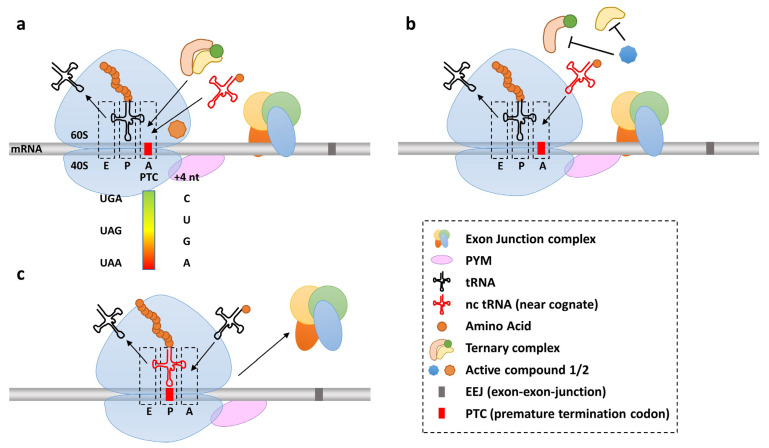
Mechanisms of nonsense suppression. (**a**) Aminoglycoside antibiotics (active compound 1 in orange) structurally render the A-site favorably for near-cognate-(nc-)tRNAs pairings. The competition between ternary release factor complex and an nc-tRNA is indicated. (**b**) Small molecules (active compound 2 in blue) that interfere with release factor binding increase the time frame for nc-tRNA pairing. (**c**) Ribosome incorporates an nc-tRNA at the premature termination codon, translation continues with release of the exon junction complex and without the induction of nonsense-mediated decay.

Aminoglycoside antibiotics and their derivatives constitute a pivotal class of readthrough-inducers [36,37,38]. Their extensive use in pre-clinical studies and in clinical trials has been reviewed [2,3] previously, and we will focus on recent developments and efforts to improve their utility. One of the most potent readthrough-inducing substances is the aminoglycoside (AG) G418 (also known as geneticin). Looking at the mechanism of action at the structural level of the ribosome, G418 and the closely related aminoglycoside gentamicin both bind to helix 44 of the 16S rRNA. Helix 44 is in close proximity to the decoding center of the ribosome and constitutes the target for many aminoglycoside antibiotics. The binding of AGs to helix 44 leads to the structural changes of the helix, shifting the position of two nucleotides, A1755 and A1756, that are essential for the decoding process. AGs bind the prokaryotic ribosome with very high affinity, but at high concentrations, G418, binds the eukaryotic ribosome, as evidenced by the crystal structures of drug-bound eukaryotic ribosomes. The ribosomal binding of AGs leads to structural changes in the A-site of the ribosome, enabling the pairing of near cognate tRNAs with the mRNA message (Figure 3a) [39,99]. Dependent on the intracellular AG concentration, translation accuracy is changed in a manner that favors SCR but also promotes drug related side effects. Due to often-unsatisfying efficiencies and marked adverse effects of the prolonged use of aminoglycosides, there is a strong demand for improved readthrough-inducing substances. In various disease contexts, large chemical libraries are screened for novel candidates. For established compounds, attempts to dissociate readthrough capacity from toxic side effects are ongoing. The most promising newly developed readthrough drug is the aminoglycoside derivative ELX-02 (also known as NB124), which has already entered phase I and II clinical trials [14,51,100,101].

### 3.2. Non-Aminoglycosides

Non-aminoglycosides represent an important class of PTC-readthrough-inducing drugs. In contrast to AGs, which preferentially bind the ribosomal A-site and lower translational fidelity, these drugs increase PTC readthrough by often-unknown mechanisms. Drugs, such as ataluren (also known as PTC124), amlexanox [102] or macrolides (e.g., azithromycin, erythromycin) potentially have the advantage of lower toxicity, mainly due to selective effects on SCR (and PTC-readthrough in particular) as opposed to lowering global translational fidelity, as seen with AGs. Although this promise was somewhat contested by a recent study by Dabrowski et al., 2021 [103], where non-aminoglycosides appeared to display lower efficiency than aminoglycosides but similar cytotoxicity.

Ever since the initial publication in 2007, ataluren (also known as PTC124) ranks among the most prominent (and probably notorious) readthrough-inducing substances. It is a synthetic non-aminoglycoside that was identified in a high content screen [104]. Shrouded in controversy, its potential to induce efficient PTC-readthrough seems highly dependent on the disease context and the experimental set-up to assess readthrough efficiencies [34,105,106,107,108,109,110]. Interestingly, ataluren and aminoglycosides seem to stimulate PTC-readthrough by orthogonal mechanisms; however, while aminoglycosides directly bind to the ribosomal decoding center and facilitate near-cognate tRNA mispairing, ataluren interferes with release factor complex (eRF1/eRF3) activity (Figure 3b) [111,112]. In an organoid model of Leber congenital amaurosis type 4 (LCA4), ataluren (10 μg/mL) induced the detectable readthrough of an *AIPL4* PTC. However, the levels of full-length protein were insufficient to rescue the disease phenotype [113]. Interestingly, an increase in ataluren concentration did not lead to an increased production of full-length protein. This type of inverted bell-shaped activity-response curve is consistent with our own (unpublished) observations and previous reports. In another recent evaluation, ataluren proved quite efficient in suppressing nonsense mutations in *Idua*, the gene associated with the progressive multisystem disorder mucopolysaccharidosis I-Hurler (MPS I-H) [114]. Specifically, the treatment of immortalized mouse embryonic fibroblast with ataluren (1–40 µM) induced the efficient readthrough of the *Idua-W402X* nonsense mutation and rescued the phenotype in vivo in a mouse model of MPS I-H. Interestingly, ataluren also exhibited a bell-shaped dose response in the respective in vitro assay (maximum response at 10 µM) [114]. Additionally, in a model for neurofibromatosis type 1 (*Nf1 R683X*), ataluren was efficacious in restoring levels of full-length protein and NF1 function (suppression of RAS effectors) in cortical neurons [115].

Importantly, a recently completed phase III clinical trial of ataluren in CF was unsuccessful [116]. This was the second large, multicenter phase III clinical trial investigating the efficacy and safety of treatment with ataluren in patients with nonsense-mutation CF (the presence of a nonsense mutation in at least one allele of the *CFTR* gene was part of the inclusion criteria). A previous phase III trial found no statistically significant improvement in pulmonary function (primary endpoint) or reduction in pulmonary exacerbations rate (secondary endpoint) upon treatment with ataluren (compared to placebo) [117]. However, some of the participants concomitantly received tobramycin (aminoglycoside antibiotic) that may have interfered with ataluren, since both substances target the eukaryotic ribosome. Post hoc subgroup analysis suggested a difference between ataluren and placebo in participants that did not receive tobramycin [117]. Therefore, an additional phase III trial was initiated in patients with nonsense-mutation CF not receiving aminoglycosides. The study enrolled 279 participants at 75 different sites in 16 countries. The study duration was 48 weeks and treatment with ataluren thrice daily. Primary endpoints (improvement in pulmonary function) and secondary endpoints (rate of pulmonary exacerbations) were assessed after 40 and 48 weeks. However, statistically significant difference between ataluren and placebo remained undetectable [116]. The results suggest that the positive signals from the post hoc subgroup analysis of the first phase III study occurred by chance and prompted the sponsor (PTC Therapeutics) to halt further clinical development of ataluren in CF caused by nonsense mutations. Potential reasons for ataluren’s failure in these clinical trials could be low bioavailability in the lungs, a lack *CFTR* mRNA due to NMD or the insertion of near-cognate tRNAs at the nonsense-codon that may affect CFTR activity. The latter scenario could be addressed by co-treatment with CFTR modulators and remains to be investigated.

On the other hand, an extended open-label phase III clinical trial in patients with nonsense mutation Duchenne muscular dystrophy (nmDMD) showed that treatment with ataluren (40 mg/kg/day) significantly delayed muscular decline and disease progression compared to standard of care alone [118]. Regardless, ataluren represents the only readthrough-inducing substance that is currently approved for clinical use. Its application for the treatment of patients suffering from DMD caused by nonsense mutations has been approved by the European Medicines Agency and an additional clinical trial is ongoing. In this trial (NCT04014530), ataluren was investigated in combination with an immune-checkpoint-inhibitor (Pembrolizumab) for the treatment of colorectal and endometrium cancers (see Supplementary Table S1). Due to deficiencies in DNA mismatch repair, these tumors bear a high mutational burden and an abundance of PTCs. The investigators reason that treatment with ataluren would facilitate the readthrough of these PTCs and consequently lead to the translation of additional out-of-frame code and the production of de novo antigenic peptides. In conjunction with PD-1 inhibition, this should amplify the anti-tumor immune response.

### 3.3. Novel Classes of Nonsense Suppressors

As described above, the efficiency of aminoglycosides to induce PTC-readthrough is highly context-dependent and varies considerably among disease models. Early on, it became apparent that the nature of the stop codon as well as the surrounding nucleotide sequence are the main determinants of readthrough efficiencies. In addition, cell- and tissue-dependent variations suggest that biological availability and effective intracellular concentration of putative readthrough-inducing substances play prominent roles in determining treatment outcomes. The uptake of aminoglycosides varies significantly between cell types. In any given disease context, the success of a putative nonsense suppression therapy is, therefore, to a considerable degree, depending on the nature of the affected tissue and the corresponding cell types and whether readthrough-promoting substances reach sufficient intracellular concentrations to exert therapeutic benefits without eliciting unacceptable side effects. Very recently, Baradaran-Heravi et al. demonstrated that TRPC cation channels influence readthrough efficiencies by controlling the cellular uptake of aminoglycosides. The inhibition of the TRPC5 channel with a small molecule (AC1903) reduced intracellular concentrations of G418 and consequently reduced the induction of PTC-readthrough in a cancer cell line (harboring a homozygous p53 nonsense mutation) and in junctional epidermolysis bullosa (JEB) patient-derived keratinocytes (harboring a homozygous mutation in *COL17A1*) [119].

Other caveats of most PTC-readthrough-inducing substances are unsatisfying efficiencies and a concomitant lack of specificity, i.e., these substances induce (desirable) readthrough of PTCs as well as (undesirable) readthrough of normal stop codons. In a recent effort to find more efficient and specific readthrough-inducing substances, Bidou et al. identified 2-guanidino-quinazoline (TLN468) by the high-throughput screening of 17,680 substances [120]. The initial screen was performed in a cell line stably expressing a secreted *Metridia* luciferase harboring a TGA premature stop codon (R213X). Hit validation was carried out using a dual reporter system. Subsequently, TLN468 was validated on more than 40 different PTCs associated with Duchenne muscular dystrophy and global readthrough activity (i.e., the readthrough of normal termination codons at the end of coding sequences) was assessed by genome-wide ribosome profiling (Ribo-seq). Strikingly, TLN468 elicited higher levels of PTC-readthrough comparable to that of gentamicin (used at a concentration of 2.5 mM) or G418 (used at a concentration of 20 µM), but at the same time induced negligible levels of readthrough of normal stop codons [120].

Similarly, 5-Fluorouridine (FUr) was identified in a data mining effort of 47,000 anti-cancer drugs as potent readthrough-inducing compound [121]. Specifically, a search of the NCI-60 database for substances that suppress growth of a subset of tumor cells carrying p53 nonsense mutations (e.g., *TP53* R213* UGA PTC) yielded 28 candidate compounds, among these the widely used anti-cancer drug 5-Fluorouracil (5-FU). Follow-up experiments revealed that the 5-FU metabolite 5-Fluorouridine (FUr) was the actual readthrough-inducing compound. FUr exerted its effect by direct incorporation into nonsense-bearing *TP53* mRNA, where it facilitated the readthrough of the PTC but not of the canonical stop codon (as shown by Ribo-seq) [121]. Tests employing mutated superfolder GFP with a UGA, UAG or UAA PTC at position 150 further demonstrated that FUr induced readthrough of UGA or UAG but not UAA.

Recently, researchers from PTC Therapeutics presented a series of guanidino quinazolines and pyrimidines scaffolds that promoted potent readthrough (*TP53* R213X UGA PTC) in a human carcinoma cell line. The researchers initially validated 2-guanidine-4-methylquinazoline (GMQ) in their experimental system. Subsequently, structure activity relationship (SAR-) guided chemical substitutions of GMQ yielded improved compounds that promoted readthrough in the nanomolar range (as low as EC_2×_ 120 nM) [122].

### 3.4. Interference with Release Factors and Translation Termination

Upon the ribosomal encounter of a PTC, the release factor complex (RFC) consisting of the eukaryotic release factors eRF1 and eRF3 mediates the release of truncated peptides. When termination at the PTC is inefficient and slow, it is possible to obtain the highest readthrough levels. This relates to the prolonged period of time the ribosome rests at the PTC without the recruitment of the RFC, which is mandatory to translation termination at PTCs as well as NTCs. Therefore, it seems likely that a reduction in eRF1 or eRF3 abundance could mediate an increase in translation readthrough at PTCs through an increased time window for near-cognate tRNA pairing. Indeed, the functional inhibition of the RFC seems to prompt the readthrough of PTCs in diverse disease contexts. Carnes et al. targeted eRF1 mRNA with siRNAs and antisense oligonucleotides in HEK293 cells stably transfected with the pAC-TMV readthrough reporter plasmid [123]. As expected, both siRNA and antisense oligonucleotide treatments significantly increased the readthrough of the UAG stop codon of the reporter construct. Further, treatments that showed the highest readthrough activity resulted in the reduction of eRF1 mRNA and eRF1 protein as supported by quantitative PCR analysis and Western blotting, respectively [123]. In other efforts to improve nonsense suppression, the small molecule degraders of eRF1, eRF3a and eRF3b were recently identified as potent enhancers of aminoglycoside-induced readthrough [124,125]. Baradaran-Heravi et al. conducted a study in several PTC disease models in the context of mucopolysaccharidosis type I-Hurler, late infantile neuronal ceroid lipofuscinosis, Duchenne muscular dystrophy and junctional epidermolysis bullosa. They used the aminoglycoside antibiotic G418 to induce translation readthrough, which was considerably enhanced by a combination of siRNAs targeted against the release factors eRF3a/b, and the application of the small molecule eRF3a degraders CC-885 and CC-90009, which induce the proteasomal degradation of eRF3a/b [124]. Moreover, eRF3 depletion reduces also eRF1 levels in conjunction with UPF1 upregulation and stabilization of TP53 nonsense transcripts indicative for NMD suppression [124]. In patient-derived immortalized keratinocytes, they achieved a remarkable increase in COL17A1 expression through the application of 100–300 µg/mL gentamicin in combination with 1 nM CC-885 or 10 or 30 nM CC-90009 in a dose-dependent manner, in comparison to gentamicin-only or CC-885/CC-90009-only treatments [124]. Importantly, the dosage used for gentamicin in this study is still relatively high compared to the level used in clinical applications (for example, therapeutic gentamicin serum concentrations are generally between 2 and 8 mg/L and maximum serum concentrations of 10–12 mg/L should not be exceeded).

In a recent example, the treatment of airway epithelial cell lines harboring nonsense mutations in the *CFTR* gene with the CRBN E3 ubiquitin ligase modulators CC-90009 (at a concentration of 0.1 µM) and SJ6986 (at a concentration of 0.2 µM) restored CFTR function to approximately 20% of the wild-type protein [126]. The combinatorial treatment of these cells with CC-90009 or SJ6986 and G418 (at a concentration of 200 µM) resulted in an astounding 50% rescue of CFTR function. The synergy of G418 and eRF3a degraders was highly context-dependent, however, since only a subset of nonsense mutations showed the synergistic effect [126]. Additionally, recently, Sharma et al. screened a huge library of 771,345 compounds in a readthrough reporter cell line. The initial screen identified 2004 compounds, which were further tested for dose-dependent activity. Among the 180 compounds that displayed dose-dependency, SRI-37240 was one of the most active. Interestingly, the co-treatment of G418 with SRI-37240 in reporter cells enhanced its activity, suggesting the independent mechanisms of nonsense suppression. In the context of cystic fibrosis, SRI-37240 (10 or 30 µM) could rescue CFTR function to a reasonable extend in cells stably expressing human CFTR cDNA containing the common G542X nonsense mutation. This effect was even more pronounced when SRI-37240 (10 µM) was combined with G418 (100 µg/mL) [125]. Western blotting confirmed a protein recovery to ~25% of mature CFTR protein in comparison to wildtype protein levels. Encouragingly, combination of G418 with SRI-37240 shows effective nonsense suppression at several other CFTR nonsense mutations tested, indicating a broader applicability to other PTC diseases. Additionally, Ribo-seq was used to estimate readthrough at canonical stop codons after the treatment with SRI-37240 in HEK293T cells and shows no increase in readthrough at canonical stop codons as compared to DMSO- and G418-treated cells [125]. For a structure-activity analysis, forty derivatives of SRI-37240 were synthesized and tested for their ability to promote readthrough and one compound, SRI-41315, exhibited better physicochemical properties. SRI-41315 demonstrated an improved readthrough activity accompanied by a dramatic reduction in eRF1, as shown by Western blotting [125].

Combined, all studies clearly indicate that interference with termination factors to increase PTC readthrough is a promising approach. Since eRF1 and eRF3 also play an important role in translation termination at NTCs, interference with these factors and translation termination in general may also affect translation termination at NTCs.

### 3.5. Ribosome Editing to Boost Production Levels of Full Length Protein from PTC mRNAs

An innovative approach targets ribosomal proteins as selective tools to change protein production levels of a protein of interest, either encoded by canonical mRNAs or by mRNAs carrying a PTC mutation [127,128]. This approach refers to the concept of specialized ribosomes and is based on the findings that subpools of heterogeneous ribosomes in different cell types and tissues exist, which preferentially translate a subset of given mRNAs [129,130]. This heterogeneity can be caused by rRNA modifications, ribosomal protein modifications and by fine-tuning ribosomal architecture by altering the functional availability of ribosomal proteins. Since the rRNAs within the functional centers of the ribosome execute the core functions of decoding and peptide bond formation during the translation of each and every mRNA, ribosomal proteins might have a more regulatory role in translation, for example, changing elongation rates and, therefore, protein production level for a given mRNA species. While the targeting of the rRNA with aminoglycosides, for example, would have a global effect on translation, due to general interference with the decoding process, ribosomal proteins could represent a promising target for specific regulation of translation. In an effort to identify ribosomal candidate proteins, which, if modulated, specifically increase the translation of a *LAMB3*-PTC (human laminin beta-3 with R635X nonsense mutation) mRNA into a full length Lamb3 protein, yeast strains carrying heterozygous deletions of ribosomal protein gene were screened [127]. These deletions demarcate differential functional availability of a given ribosomal protein, thus generating a subclass of “specialized ribosomes”. Indeed, ribosomal protein rpL35 was identified a ribosomal target to boost production levels of full length Lamb3 but not of unrelated proteins encoded by a PCT mRNA. Furthermore, two small molecule binders of rpL35 were identified bioinformatically, each one with FDA approval for a different application (repurposable drugs). Their binding to rpL35 in solution was demonstrated by NMR analysis [128]. Now, studies are under way to study the effect of treatment with rpL35 ligands on boosting expression levels of full length Lamb3 encoded by a PTC mRNA. If successful, this would allow the development of a customized systemic therapy for the rare skin disease junctional epidermolysis bullosa, which is predominantly caused by homozygosity for PCT mutations in the skin anchor protein Lamb3.

## 4. Nonsense Suppression in the Context of Rare Genetic Diseases and Cancer

### 4.1. Cystic Fibrosis

A major obstacle to successful readthrough therapy is the instability of PTC-bearing mRNAs due to nonsense-mediated mRNA decay (NMD). Even the highly efficient induction of translational readthrough can therefore be therapeutically insignificant in light of the very low availability of PTC-bearing mRNAs. In cystic fibrosis (CF), certain N-terminal PTCs (such as E60X, L88X) result in the production of partially functional cystic fibrosis transmembrane conductance regulator (CFTR) proteins due to downstream translation initiation. Messages containing these 5′ nonsense variants seemed to evade NMD. The production of full length CFTR was efficiently induced in primary human nasal epithelial cells by treatment with G418 (at concentrations of 100 µM and 400 µM) or ELX-02 (at concentrations of 100 µM and 200 µM). Importantly, combinatorial treatment with CFTR modulators was obligatory for achieving functional CFTR reconstitution, since treatment with readthrough inducers alone produced misfolded CFTR [131]. Another recent evaluation of ELX-02 in rare *CFTR* nonsense mutations revealed a favorable “U UGA C” sequence context for *CFTR* mRNA stabilization and readthrough [14], while the co-treatment of patient-derived human nasal epithelial cells with ELX-02 (1 mM) and an NMD inhibitor (SMG1i, 5 µM) increased *CFTR* mRNA levels compared to ELX-02 treatment alone, it did not result in significant increases in CFTR activity [14]. Recent studies in PTC-containing intestinal organoids further underscore the promise of combinatorial treatment in rescuing CFTR function [101]. Most efficient rescue of CFTR function was achieved for most donors with a combination treatment consisting of a readthrough-inducing compound (ELX-02), NMD inhibitor (by SMG1i) and substances targeting CFTR function (two CFTR correctors, VX-445 and VX-661, and the CFTR potentiator VX-770). Although the described combinatorial approach is geared predominantly toward the treatment of CF, the overall implications apply to other diseases caused by nonsense mutations and demonstrate its use as a viable option for nonsense suppression in general.

### 4.2. Retinitis Pigmentosa

In fibroblasts of patients with retinitis pigmentosa, caused by homozygous nonsense mutations (R523X) in the gene *FAM161A*, treatment with ataluren or gentamicin induced the partial re-expression of full-length protein and restored ciliogenesis in these cells. Interestingly, ataluren (at a concentration of 10 µg/mL) and gentamicin (at concentration of 1 mg/mL) restored similar levels of full-length protein in patient cells, but ataluren was more efficient in restoring ciliary function [132].

### 4.3. Shwachman–Diamond Syndrome

In bone marrow stem cells from patients with Shwachman–Diamond syndrome (a rare inherited disease of the pancreas and bone marrow that entails an increased risk of developing acute myeloid leukemia at an early age), ataluren slightly increased levels of SBDS protein (the loss of which was caused the K62X mutation). Furthermore, treatment with ataluren (at a concentration of 2.5 µM) or an optimized analog (NV848, at concentrations of 1–10 µM) caused the detectable re-expression of SBDS protein (assessed by Western blot densitometry analysis) in hematopoietic (primary peripheral blood mononuclear cells) and non-hematopoietic cells (periodontal ligament stem cells) from patients [133].

### 4.4. Alport Syndrome

A comprehensive NanoLuc-based investigation of known readthrough inducers in the context of a larger number of nonsense mutations in *COL4A5*—the gene involved in Alport syndrome—suggested the applicability of PTC readthrough therapy for a subset of mutations (at least 11 out of 49 nonsense mutations in *COL4A5*) [134]. A NanoLuc-based translational reporter system was used to evaluate different readthrough inducers on 49 different nonsense mutations at different concentrations and in four different cell types. While G418 showed the highest induction of readthrough, ELX-02 (100–300 µg/mL) and 2,6-diaminopurine (25–300 µM) also proved to be efficient. On the contrary, treatment with ataluren (20–50 µM) or RTC14 (25–100 µM) resulted in no detectable induction. Furthermore, combinatorial treatment with the antimalarial drug mefloquine appeared to enhance the G418- and gentamicin-induced readthrough of a nonsense mutation (R1563X) [134].

### 4.5. Breast Cancer

Since inherited or de novo nonsense mutations in tumor suppressor genes lead to loss-of-function and the rise of cancer, nonsense suppression may represent an attractive treatment option. For example, naturally occurring nonsense mutations in the tumor suppressor gene BRCA1 are associated with an increased risk of breast cancer. Recently, Abreu et al. confirmed the ability of the aminoglycoside G418 to induce the production of full-length protein and restore BRCA1 function in a human breast cancer cell line. Looking at different clinically relevant nonsense mutations, they also found context-dependent readthrough efficiencies [135].

### 4.6. Epidermolysis Bullosa

The ability of different readthrough inducers to restore the expression of full-length COL17 protein in keratinocytes from patients with junctional epidermolysis bullosa was recently assessed by Has et al. The researchers focused their attention on a subset of patients carrying homozygous nonsense mutations in the *COL17A1* gene (p.W464*, p.R688*, p.R1226*, p.S140*). Interestingly, treatment with ataluren and amlexanox failed to elicit the production of detectable levels (by Western blot) of full-length COL17 proteins. The response to aminoglycosides gentamicin, paromomycin and G418 depended on the nature of the stop codon. Although some mutations (e.g., p.W464*) displayed a robust response, other mutations (i.e., p.R1226* and p.S140*) were completely refractory to aminoglycoside treatment [136].

A recent, single-patient evaluation of intravenously administered gentamicin for the treatment of epidermolysis bullosa simplex with muscular dystrophy (EBS-MD) yielded promising results [137]. In the affected patient, a homozygous nonsense mutation in the *PLEC1* gene (c.4261C>T, p.Q1421*, UAG PTC) lead to almost undetectable levels of plectin at the dermoepidermal junction. Tests in cultured primary keratinocytes from the patient suggested that gentamicin (400 µg/mL) or G418 (25 μg/mL) treatment promoted the expression of full-length plectin. Consequently, the patient was treated for 14 days with intravenous gentamicin (7.5 mg/kg/day) and the clinical outcome—including plectin expression at the dermoepidermal junction—was assessed [137]. Although, gentamicin treatment led to increased plectin levels (during the 5-month follow-up), other clinical parameters, such as skeletal and respiratory muscle function, showed only marginal improvements. Considering the patient’s perceived increase in quality of life upon gentamicin treatment, the overall results of this case study further promote cautious optimism when it comes to the systemic administration of gentamicin as a treatment option for severe forms of EB.

## 5. Conclusions

Ever since the first report of induced PTC-readthrough in the context of a genetic disease in 1996, there has been a continuous effort to advance nonsense suppression therapy into clinical settings for a growing number of conditions. The development of aminoglycosides into more efficient and less toxic analogs is rapidly progressing, as is the search for non-aminoglycoside alternatives. Large-scale screening efforts unearthed novel compounds with superior readthrough-inducing properties in experimental phases. In addition, it became evident that readthrough drugs show cell and tissue specificity. Consequently, due to the molecular complexities of readthrough induction, preclinical results did and do not always seamlessly translate into successful clinical trials and suggest that more personalized strategies are warranted, for example, ribosome editing. Nonetheless, novel classes of readthrough-inducing compounds, such as small molecules that interfere with translation termination and release factor function, hold great promise. In particular, the combinatorial treatment of established readthrough inducers with these novel compounds and/or substances that increase the cellular uptake of aminoglycosides and their derivatives is alluring. The prospect of personalized, context-dependent nonsense suppression with increased efficacy and concomitantly decreased toxic side effects may lead to the broad applicability of this attractive therapeutical avenue in a large spectrum of genetic disorders.

## Data Availability

Not applicable.

## Supplementary Materials

The following supporting information can be downloaded at: https://www.mdpi.com/article/10.3390/ijms24076101/s1.

## Abbreviations

AG	aminoglycoside
CBC	cap binding complex
CF	cystic fibrosis
CFTR	cystic fibrosis transmembrane conductance regulator
DECID	decay-inducing complex
DMD	Duchenne muscular dystrophy
EB	epidermolysis bullosa
EJC	exon junction complex
eRF1	eukaryotic release factor 1
JEB	junctional epidermolysis bullosa
NMD	nonsense mediated mRNA decay
NTC	normal termination codon
PTC	premature termination codon
RFC	release factor complex
RPFs	ribosome-protected fragments
SCR	stop codon readthrough
TRIDs	translational-readthrough-inducing drugs
