# Supplementary material for: Emerging Personalized Opportunities for Enhancing Translational Readthrough in Rare Genetic Diseases and Beyond
Source: Int J Mol Sci. 2023 Mar 23;24(7):6101. doi: 10.3390/ijms24076101 (PMC10093890; doi:10.3390/ijms24076101)
Supplement: Supplementary file 1 [file ijms-24-06101-s001.zip › Suppl_table_clinicaltrials.pdf]

Supplementary Table S1. Interventional clinical trials with translation readthrough-inducing drugs (TRIDs).

NCT Number	Title	Status	Conditions	Interventions	Phases	Start Date	Completion Date
**NCT05448755**	A Study of ELX-02 in Patients With Alport Syndrome	Recruiting	Alport Syndrome	Drug: ELX-02	Phase 2	November 28, 2022	May 30, 2023
**NCT01141075**	Ataluren for Nonsense Mutation Methylmalonic Acidemia	Terminated	Amino Acid Metabolism, Inborn Errors	Drug: Ataluren	Phase 2	July 19, 2010	November 3, 2011
**NCT04117880**	A Phase 2 Open Label Extension Study in Participants With Nonsense Mutation Aniridia	Withdrawn	Aniridia	Drug: Ataluren	Phase 2	December 31, 2018	January 31, 2021
**NCT02647359**	Study of Ataluren in Participants With Nonsense Mutation Aniridia	Completed	Aniridia	Drug: Ataluren|Drug: Placebo	Phase 2	January 31, 2016	January 22, 2021
**NCT04014530**	Pembrolizumab With Ataluren in Patients With Metastatic pMMR and dMMR Colorectal Carcinoma or Metastatic dMMR Endometrial Carcinoma: the ATAPEMBRO Study	Recruiting	Colorectal Cancer|Endometrium Cancer	Drug: Ataluren + Pembrolizumab	Phase 1|Phase 2	August 1, 2019	August 1, 2023
**NCT02107859**	Study of Ataluren (PTC124) in Cystic Fibrosis	Terminated	Cystic Fibrosis	Drug: Ataluren	Phase 3	May 23, 2014	June 5, 2017
**NCT02456103**	Extension Study of Ataluren in Participants With Nonsense Mutation Cystic Fibrosis	Terminated	Cystic Fibrosis	Drug: Ataluren	Phase 3	August 31, 2015	June 2, 2017
**NCT01140451**	Extension Study of Ataluren (PTC124) in Cystic Fibrosis	Completed	Cystic Fibrosis	Drug: Ataluren	Phase 3	August 12, 2010	December 2, 2013

**NCT03256968**	PTC Study to Evaluate Ataluren in Combination With Ivacaftor	Completed	Cystic Fibrosis	Drug: Ataluren	Phase 4	January 27, 2017	December 31, 2018
**NCT00458341**	A Study of Ataluren in Pediatric Participants With Cystic Fibrosis	Completed	Cystic Fibrosis	Drug: Ataluren	Phase 2	March 23, 2007	February 29, 2008
**NCT00237380**	Safety and Efficacy of Ataluren (PTC124) for Cystic Fibrosis	Completed	Cystic Fibrosis	Drug: Ataluren	Phase 2	November 30, 2005	May 31, 2006
**NCT02139306**	Study of Ataluren in Nonsense Mutation Cystic Fibrosis (ACT CF)	Completed	Cystic Fibrosis	Drug: Ataluren (PTC124)|Drug: Placebo	Phase 3	August 1, 2014	November 1, 2016
**NCT00803205**	Study of Ataluren (PTC124) in Cystic Fibrosis	Completed	Cystic Fibrosis	Drug: Ataluren|Drug: Placebo	Phase 3	September 8, 2009	November 12, 2011
**NCT04126473**	A Phase 2 Study to Evaluate the Safety, Tolerability, PK and PD in Cystic Fibrosis Patients With at Least 1 G542X Allele	Recruiting	Cystic Fibrosis	Drug: ELX-02|Drug: Ivacaftor	Phase 2	November 5, 2019	July 31, 2022
**NCT04135495**	A Phase 2 Study to Evaluate the Safety, Tolerability, PK and PD of ELX-02 in Cystic Fibrosis Patients With G542X Allele	Completed	Cystic Fibrosis	Drug: ELX-02|Drug: Ivacaftor	Phase 2	November 25, 2019	October 3, 2022
**NCT00376428**	Interest of Gentamicin-induced Readthrough in Cystic Fibrosis Patients	Terminated	Cystic Fibrosis	Drug: Gentamicin	Phase 2	January 2003	June 2005
**NCT03256799**	Evaluation of Ivacaftor in Patients Using Ataluren for Nonsense Mutations	Completed	Cystic Fibrosis	Drug: Ivacaftor/Ataluren	Phase 4	March 17, 2017	February 16, 2018

**NCT00351078**	PTC124 for the Treatment of Cystic Fibrosis	Completed	Cystic Fibrosis	Drug: PTC124	Phase 2	December 2006	July 2007
**NCT00234663**	PTC124 for Cystic Fibrosis	Completed	Cystic Fibrosis	Drug: PTC124	Phase 2	September 1, 2005	August 1, 2006
**NCT00005574**	Gentamicin Treatment of Muscular Dystrophy	Completed	Duchenne Muscular Dystrophy	Drug: Gentamicin	Phase 1	February 2000	January 2001
**NCT03648827**	A Study to Assess Dystrophin Levels in Participants With Nonsense Mutation Duchenne Muscular Dystrophy (nmDMD)	Completed	Duchenne Muscular Dystrophy	Drug: Ataluren	Phase 2	December 21, 2018	October 23, 2020
**NCT03796637**	A Study to Assess Dystrophin Levels in Participants With Nonsense Mutation Duchenne Muscular Dystrophy (nmDMD) Who Have Been Treated With Ataluren	Completed	Duchenne Muscular Dystrophy	Drug: Ataluren	Phase 2	April 11, 2019	June 3, 2019
**NCT02819557**	Study of Ataluren in ≥2 to <5 Year-Old Male Participants With Duchenne Muscular Dystrophy	Completed	Duchenne Muscular Dystrophy	Drug: Ataluren	Phase 2	June 9, 2016	February 9, 2018
**NCT01247207**	Study of Ataluren in Previously Treated Participants With Nonsense Mutation Dystrophinopathy (nmDBMD)	Enrolling by invitation	Duchenne Muscular Dystrophy	Drug: Ataluren	Phase 3	November 30, 2010	December 15, 2023
**NCT00759876**	Phase 2a Extension Study of Ataluren (PTC124) in	Terminated	Duchenne Muscular Dystrophy	Drug: Ataluren	Phase 2	August 13, 2008	May 17, 2010

	Duchenne Muscular Dystrophy (DMD)						
**NCT00451074**	Six Month Study of Gentamicin in Duchenne Muscular Dystrophy With Stop Codons	Completed	Duchenne Muscular Dystrophy	Drug: Gentamicin infusions twice a week for six months	Phase 1	March 2007	July 2009
**NCT00264888**	Safety and Efficacy Study of PTC124 in Duchenne Muscular Dystrophy	Completed	Duchenne Muscular Dystrophy	Drug: PTC124	Phase 2	December 2005	May 2007
**NCT00847379**	Phase 2B Extension Study of Ataluren (PTC124) in Duchenne/Becker Muscular Dystrophy (DMD/BMD)	Terminated	Duchenne Muscular Dystrophy	Drug: Ataluren	Phase 2	January 31, 2009	May 24, 2010
**NCT01009294**	Study of Ataluren (PTC124) in Nonambulatory Participants With Nonsense-Mutation-Mediated Duchenne/Becker Muscular Dystrophy (nmDMD/BMD)	Terminated	Duchenne Muscular Dystrophy	Drug: Ataluren|Drug: Chronic Corticosteroid Therapy	Phase 2	January 13, 2010	March 23, 2010
**NCT00592553**	Phase 2B Study of PTC124 (Ataluren) in Duchenne/Becker Muscular Dystrophy (DMD/BMD)	Completed	Duchenne Muscular Dystrophy	Drug: Ataluren|Drug: Placebo	Phase 2	February 29, 2008	December 31, 2009
**NCT01557400**	Study of Ataluren for Previously Treated Participants With Nonsense Mutation Duchenne/Becker Muscular Dystrophy	Completed	Duchenne Muscular Dystrophy	Drug: Ataluren	Phase 3	May 20, 2012	January 19, 2018

	(nmDBMD) in Europe, Israel, Australia, and Canada						
**NCT01918384**	Phase II Study of NPC-14 (Arbekacin Sulfate) to Explore Safety, Tolerability, and Efficacy in Duchenne Muscular Dystrophy	Unknown status	Duchenne Muscular Dystrophy	Drug: NPC-14|Drug: Placebo	Phase 2	August 1, 2013	October 2015
**NCT03179631**	Long-Term Outcomes of Ataluren in Duchenne Muscular Dystrophy	Active, not recruiting	Duchenne Muscular Dystrophy	Drug: Ataluren|Drug: PLACEBO	Phase 3	July 6, 2017	July 20, 2023
**NCT02090959**	An Extension Study of Ataluren (PTC124) in Participants With Nonsense Mutation Dystrophinopathy	Terminated	Duchenne Muscular Dystrophy	Drug: Ataluren	Phase 3	March 20, 2014	June 12, 2018
**NCT01826487**	Phase 3 Study of Ataluren in Participants With Nonsense Mutation Duchenne Muscular Dystrophy (nmDMD)	Completed	Duchenne Muscular Dystrophy	Drug: Ataluren|Drug: Placebo	Phase 3	March 26, 2013	August 20, 2015
**NCT04336826**	A Study to Evaluate the Safety and Pharmacokinetics of Ataluren in Participants From ≥6 Months to <2 Years of Age With Nonsense Mutation Duchenne Muscular Dystrophy (nmDMD)	Recruiting	Duchenne Muscular Dystrophy	Drug: Ataluren	Phase 2	December 29, 2021	March 31, 2023
**NCT04644627**	Topical Gentamicin Nonsense Suppression Therapy of EB	Completed	Epidermolysis Bullosa	Drug: Gentamicin Sulfate	Phase 1|Phase 2	December 1, 2020	May 1, 2022

**NCT02758626**	Ataluren for Nonsense Mutation in CDKL5 and Dravet Syndrome	Completed	Epilepsy	Drug: ataluren|Drug: Placebo	Phase 2	November 1, 2016	February 27, 2021
**NCT04454151**	Azithromycin Treatment for Readthrough of APC Gene Stop Codon Mutations in Familial Adenomatous Polyposis	Unknown status	Familial Adenomatous Polyposis	Drug: Azithromycin Tablets	Phase 4	August 1, 2020	April 1, 2022
**NCT02175914**	Erythromycin Treatment for Readthrough of APC Gene Stop Codon Mutations in Familial Adenomatous Polyposis	Unknown status	Familial Adenomatous Polyposis	Drug: Erythromycin	Phase 4	June 2014	June 2016
**NCT02354560**	Erythromycin Treatment for Readthrough of APC Gene Stop Codon Mutation in Familial Adenomatous Polyposis-minors' Adjusted Version	Unknown status	Familial Adenomatous Polyposis	Drug: Erythromycin	Phase 4	June 2015	December 2017
**NCT03309605**	Phase 1 Study of ELX-02 in Healthy Adult Subjects	Completed	Healthy	Drug: ELX-02|Drug: Placebo	Phase 1	October 11, 2017	July 17, 2019
**NCT03292302**	Phase 1 Study of ELX-02 in Healthy Adults	Completed	Healthy	Drug: Placebo|Drug: ELX-02	Phase 1	September 26, 2017	December 15, 2017
**NCT02807961**	Safety, Tolerability, Pharmacokinetics of ELX-02 in Healthy Adult Volunteers	Terminated	Healthy	Drug: ELX-02|Drug: Placebo	Phase 1	July 2016	May 2017
**NCT02409004**	Effects of Rifampin on the Pharmacokinetics of Ataluren	Completed	Healthy	Drug: Ataluren|Drug: Rifampin	Phase 1	February 2015	March 2015
**NCT00947193**	Study of Ataluren (PTC124) in Hemophilia A and B	Terminated	Hemophilia A|Hemophilia B	Drug: Ataluren	Phase 2	October 14, 2009	August 30, 2011

**NCT03492866**	Efficacy of Topical Gentamycin for Hereditary Hypotrichosis Simplex Caused by Nonsense Mutations in CDSN	Unknown status	Hereditary Hypotrichosis Simplex	Drug: Gentamicin Sulfate	Phase 2	April 1, 2018	April 1, 2019
**NCT03776539**	A Study to Evaluate the Effects of Renal Impairment on the Pharmacokinetics of ELX-02	Completed	Impaired Renal Function	Drug: ELX-02	Phase 1	January 4, 2019	August 7, 2019
**NCT03526159**	Gentamicin for Junctional Epidermolysis Bullosa	Recruiting	Junctional Epidermolysis Bullosa	Drug: Gentamicin Sulfate	Phase 1|Phase 2	June 1, 2018	August 31, 2020
**NCT04140786**	Optimizing IV Gentamicin in JEB	Recruiting	Junctional Epidermolysis Bullosa	Drug: Gentamicin Sulfate, Injectable	Phase 1|Phase 2	October 31, 2019	November 1, 2023
**NCT04069260**	A Phase 2 Study of ELX-02 in Patients With Nephropathic Cystinosis	Terminated	Nephropathic Cystinosis	Drug: ELX-02	Phase 2	August 2, 2019	December 17, 2019
**NCT03392909**	Intravenous Gentamicin Therapy for Recessive Dystrophic Epidermolysis Bullosa (RDEB)	Recruiting	Recessive Dystrophic Epidermolysis Bullosa	Drug: Gentamicin	Phase 1|Phase 2	July 5, 2018	December 1, 2023
**NCT03012191**	Gentamicin for RDEB	Completed	Recessive Dystrophic Epidermolysis Bullosa	Drug: Gentamicin Sulfate	Phase 1|Phase 2	February 2, 2017	August 31, 2018
**NCT02698735**	Gentamicin Therapy for Recessive Dystrophic Epidermolysis Bullosa (RDEB) Nonsense Mutation Patients	Completed	Recessive Dystrophic Epidermolysis Bullosa	Drug: Gentamicin|Drug: Placebo	Phase 1|Phase 2	February 25, 2016	June 30, 2017
